# 

**Published:** 1903-02-14

**Authors:** 


					the hospital. The standard of highest purity."?Lancet. Feb. hth, 1903
x CADBURY'S ^ |j jg, CADBURY'S
COCOA v \1^ Jr^ WA COCOA
ABSOLUTELY PURE. HO DRUGS OR ALKALIES USED.
LaspoiT Wes tern bifiBUABr
No. 855/Yol. XXXIII. LfSGSSifl Saturday,
Feb. 14th, 1903.
[Subscripti?n^Term?,J pRI0H TWOPENCE.

				

